# Mapping the Instruments Used to Measure Well‐Being on Children With Disabilities: A Scoping Review

**DOI:** 10.1111/cch.70142

**Published:** 2025-09-11

**Authors:** Estrella Torres Cabo, Mats Granlund, Magnus Ivarsson

**Affiliations:** ^1^ CHILD, School of Education and Communication Jönköping University Jönköping Sweden; ^2^ CHILD, School of Health and Welfare Jönköping University Jönköping Sweden; ^3^ Department of Mental Health Norway's Technical and Natural Science University Trondheim Norway; ^4^ Department of Behavioral Sciences and Learning Linköping University Linköping Sweden

**Keywords:** children with disabilities, cognitive accessibility, flourishing, measures, mental health, well‐being

## Abstract

**Objective:**

To describe the self‐report instruments used to measure well‐being in children with disabilities, investigate their psychometric quality, cognitive accessibility and alignment with Keyes's operationalization of well‐being, including emotional, psychological and social aspects.

**Methods:**

MEDLINE, ProQuest, PubMed and CINAHL were searched for articles published from 2011 to March 2023, identifying 724 studies. Synonyms provided by thesaurus on the main constructs: ‘children’, ‘measure’, ‘disability’ and ‘mental health’ were employed in the search strategy. Two reviewers independently screened articles using criteria following the SPIDER framework, resulting in the inclusion of 13 articles. From these, 10 instruments were extracted and critically appraised using the COSMIN checklist, evaluating their psychometric properties. The cognitive accessibility and alignment to Keyes's dual continua model of mental health were analysed.

**Results:**

The included instruments had fair to excellent psychometric properties. Instruments designed for children with disabilities demonstrated greater cognitive accessibility compared with those for the general child population. Well‐being was primarily identified as hedonic, with social aspects of positive functioning underrepresented. Five of the 10 instruments measured quality of life but were used as well‐being measures in studies. While most items aligned with the dual continua model, no single instrument encompassed all three aspects of well‐being.

**Conclusions:**

Although there is a growing effort to include children with disabilities in well‐being research, a consensus on a comprehensive well‐being self‐report measure is lacking. Further research is needed to develop a multidimensional operationalization that includes psychological and social aspects of well‐being for children with disabilities.

**Summary:**

- Well‐being measures focus on hedonic aspects—emotional well‐being—and often exclude the social aspect of positive functioning within eudaimonia.
- There is a conceptual overlap between quality‐of‐life measures and well‐being measures.
- There is a tendency to emphasize individual aspects of well‐being over social dimensions.
- Participatory research methods reflect the ecological validity of the construct of cognitive accessibility as proposed by Kramer and Schwarts.
- Keyes' MHC‐SF remains the most global proposal integrating eudemonic and hedonic aspects of well‐being but has not been validated in children with disabilities.

## 1 Introduction

Recent research involving adolescents and adults suggests that well‐being is better conceptualized as a distinct, multifaceted construct/continuum for positive functioning, rather than the positive endpoint of a unidimensional mental health concept (Keyes 2002). Extending this dual continuum model of mental health to children with disabilities introduces methodological and conceptual challenges. For example, when assessing well‐being, some instruments may not be adapted to the specific needs of this population, hindering reliable self‐reporting. Additionally, it remains unclear to what extent measures developed in line with the dual continuum framework are being applied in studies involving children with disabilities. The present study sought to fill these gaps in the literature by reviewing self‐report instruments used to measure well‐being in children with disabilities, how those instruments were adapted to the needs of children with disabilities and how the content relates to the dual continua model of mental health.

This work acknowledges the importance of including people with disabilities in all matters affecting their lives, as called for by the United Nations Convention on the Rights of People with Disabilities (UNCRPD; United Nations [UN] 2006). Such effort converges with the promotion of the well‐being of all children and adolescents, which numerous countries and organizations are already actively engaged in, aligning with the United Nations' Sustainable Development Goal 3. Including the perspective of the child in studies concerning the well‐being of children with disabilities is not only a question of principles of inclusion but also of validity (Granlund et al. 2021). Further, measuring well‐being in children with disabilities with self‐rating instruments requires the use of measures that are understood and possible to use by the children.

Conceptual clarity relating to well‐being and mental health problems is essential when involving children with disabilities. Disability, as defined in the International Classification of Functioning, Disability, and Health (WHO 2001), is conceptualized as an umbrella term for difficulties with functioning on body, activity or participation levels in the interaction with the environment. By using a profile approach including body as well as activity and participation, a comprehensive view of a person's functioning can be generated. Children with disabilities have difficulties with body functions and/or activity performance that hinder their functioning in everyday life. Mental health problems can be conceptualized as challenges in function linked to emotion or behaviour (e.g., anxiety or difficulties getting along with peers) that may or may not meet the criteria of a formal diagnosis (Granlund et al. 2021; Wissow et al. 2008). In the Diagnostic and Statistical Manual of Mental Disorders (5th ed.; DSM–5; American Psychiatric Association 2013) and the International Statistical Classification of Diseases and Related Health Problems (11th ed.; ICD‐11; World Health Organization 2019), both diagnoses that are commonly thought of in terms of psychopathology (such as depression) and disabilities (such as neurodevelopmental disorders) are listed. If diagnoses that tend to be perceived as psychopathology and disabilities are not explicitly delimited from each other, there is a risk that people with neurodevelopmental disorders are judged to exhibit higher degrees of mental health problems than they actually have. Well‐being can be defined as positive functioning and positive feelings; in the dual continua model, well‐being is separated from mental health problems and is seen as a continuum of its own (Keyes et al. 2002). The dual continua model reduces the risk of bias. Within this framework, it is possible to acknowledge that individual differences in the experience of well‐being are not a logical consequence of a diagnosis. Thus, children can experience well‐being and mental health problems simultaneously.

Based on the dual continua model and the World Health Organization's (WHO; 2005) definition of mental health, the Mental Health Continuum Scale—Short Form (MHC‐SF; Keyes 2002) was developed. The dual continua model defines mental health as a multidimensional construct that includes positive affect (emotional well‐being) and positive functioning (psychological and social well‐being; Keyes 2002). The model integrates the hedonic and eudaimonic philosophical traditions, relating well‐being to both experiences of positive emotions, happiness, interest in new things, life satisfaction (Keyes 2002) and being involved in a goal‐directed or meaningful life (based on Ryan and Deci 2001). This approach acknowledges that individuals can experience high states (flourishing) or low states of positive mental health (languishing) irrespective of the presence of mental problems (Westerhof and Keyes 2010). Consequently, it is possible to experience flourishing in the presence of severe mental health problems (Keyes 2002). Studying well‐being within the dual continua model provides a holistic view beyond the absence of problems, acknowledging functional challenges related to behaviour or emotions affecting daily life without leading to a mental health diagnosis (Wissow et al. 2008). The use of positive mental health measures when assessing children emphasizes the role of positive factors shaping their well‐being rather than only the absence of problems (Morrison and Kirby 2010). Following the same line of reasoning, participation has a stronger relation to well‐being, as measured by MHC‐SF (Keyes 2002), than to mental health problems (Augustine et al. 2022).

### 1.1 Measuring Mental Health in Children With Disabilities

While originally designed for adults (Keyes 2006), the MHC‐SF has later been validated in adolescents (Guo et al. 2015; Keyes 2006) and youth in primary care mental health services (Donnelly et al. 2019). However, the psychometric properties of the scale have never been investigated in children younger than 12 years of age, partly because of concerns with cognitive accessibility.

Cognitive accessibility recognizes the link between the functional demands intrinsic to a self‐report scale and administration procedure, and the respondent's functional capacity in responding to such scales (Rios et al. 2016; Kramer and Schwartz 2017). Previous research has shown that an increase in perceptual and sensory demands (e.g., the font type, size and style, and line length), as well as motor demands (Dolan et al. 2010; Beddow 2012), makes it increasingly difficult to manage higher‐level cognitive functions (Clark et al. 2006). Considering this aspect of the design and administration procedure of the measures as well as environmental characteristics and the child's current health will optimize the interaction between the respondent and the item (Fujiura 2012; Finlay and Lyons 2001; seen in Kramer and Schwartz 2017) and facilitate the participation of those often excluded from self‐rating–based surveys, such as young children and children with disabilities (Adair et al. 2018). As of today, proxy ratings are prevalent in studies of mental health‐related problems in children with disabilities (Adair et al. 2018) albeit evidence suggests a limited consensus between proxy and child reports on subjectively experienced phenomena (Davis et al. 2007; Achenbach et al. 1987; De Los Reyes et al. 2015). It can be argued that some concepts, such as well‐being, are inherently subjective and cannot be validly assessed by an external observer; therefore, self‐report instruments are important. Conceptual agreement on the multidimensional nature of children's well‐being is marred by ambiguity on its specific dimensions (O'Hare and Gutierrez 2012), pronounced by a conceptual lack of inclusivity of children with disabilities (Granlund et al. 2021). Hence, it is important not only to consider a conceptualization of mental health that makes space for the well‐being of people with disabilities but also to implement self‐report measures that do not constrain their inclusion.

## 2 Aims

This study aimed to identify instruments currently used for assessing self‐reported well‐being in children with disabilities, as well as assessing their quality, cognitive accessibility and alignment with Keyes's (2006) operationalization of the dual continua model of mental health.

## 3 Methods

### 3.1 Search Strategy

Adhering to the PRISMA‐ScR guidelines (Tricco et al. 2018), a comprehensive literature search was conducted on the electronic databases PubMed, MEDLINE, CINAHL and ProQuest. Different search terms were identified for the four main constructs (‘mental health’, ‘measure’, ‘child’ and ‘disability’). For example, the search terms relating to children encompassed ‘adolescence’, ‘adolescents’, ‘child’ or ‘teenager’ (the full list of search terms used is available at the linked repository DOI 10.17605/OSF.IO/VX2TG). Filters for language (English), age group (child) and publication date (1 January 2010–31 March 2023) were applied. The publication time was limited to 2010–2023 for two reasons: (1) the focus of the study was on instruments that are currently in use and their relation to the three domains in Keyes' (2006) well‐being model, and (2) the number of citations to the authors of the Mental Health Continuum Model (Westerhof and Keyes 2010) spikes and continues to rise after publishing the model in the Keyes and Westerhof article, according to Google Scholar.

### 3.2 Selection Criteria

The SPIDER (Cooke et al. 2012) framework was used when defining the eligibility criteria for the studies identified in the searches (see Table 1). Studies measuring well‐being with self‐report instruments in children and teenagers with a health‐related condition and/or disability were included in the study.

**TABLE 1:** Exclusion and inclusion criteria following SPIDER.

	Inclusion	Exclusion
Sample (S)	Children and adolescents (age 6–17 years) with a chronic health‐related condition or disorder that can be detected or diagnosed during the developmental period.	Studies with samples consisting exclusively of children under 6 years of age or adults (i.e., aged 18 years or more). Studies with samples that exclusively encompass children without disability.
Phenomenon of interest (PI)	Self‐report scales measuring well‐being. The instrument is used to measure well‐being, not mental problems.	Studies with instruments that are described to measure related but quantitatively different outcomes such as mental health problems or quality of life. Articles that do not mention and/or provide a reference to the instrument. Be it to a manual, original article creating the instrument or validation study.
Design of study (D)	Quantitative studies	Meta‐analysis or systematic reviews. Qualitative studies
Evaluation (E)	Not related to the search	
Research type (R)	Not related to the search	

The goal was to identify studies that measured well‐being with an instrument designed for the purpose; therefore, studies that used items of surveys or scales that could not be linked to a psychometric study of its properties were excluded. Studies with children aged 5 years or younger were excluded because it is unreasonable to expect self‐rating of well‐being in children of that age.

Studies had to include children with health‐related conditions or disabilities arising during the developmental period, encompassing a range of long‐term conditions characterized by limitations in mental, sensory and/or physical functioning (World Health Organization 2013; Center for Disease Control and Prevention 2013) such as intellectual disability (ID), cerebral palsy, autism spectrum disorder and speech sound disorders. Included were also all diagnoses categorized as neurodevelopmental disorders in the DSM‐5.

### 3.3 Study Selection

The records identified through the search were uploaded to Rayyan (Ouzzani et al. 2016). The eligibility screening procedure was conducted by two independent researchers with an inter‐rating agreement of 98.3%. Disagreement was discussed, reaching consensus after taking the pre‐established inclusion and exclusion criteria as the base reference.

### 3.4 Instrument Quality Assessment

The validity of instruments is important for their validity of assessment, therefore the quality of the included instruments was assessed following the COSMIN Checklist (Mokkink et al. 2010) despite doing a scoping review. A study of the psychometric properties of the scale in the specific sample (children with disabilities) was sought for retrieval. Six out of 12 of the ‘boxes’ in the COSMIN checklist were deemed relevant for the present study: (A) Internal consistency, (B) Reliability, and relative measures, (C) Measurement error, (D) Content validity, (E) Structural validity and (F) Hypotheses testing (construct validity). As the instructions dictate, the final score was assessed using the boxes mentioned in the psychometric paper, and the overall quality of the instruments was determined by one author (‘excellent’, ‘good’, ‘fair’ and ‘poor’) based on the lowest‐rated box (Mokkink et al. 2010). Even though it was not the main focus, including the quality appraisal of the instruments was deemed needed to disclose their potential limitations and contextualize the findings. The quality appraisal grounds other findings related to the instruments, in this case on accessibility and construct limitations. Moreover, it allows us to ensure the appropriateness of the measures across fields of research in which the target group is not necessarily normative adults. These aspects have been previously mentioned by other researchers (e.g., Maguire et al. 2023 or Bentley et al. 2019).

### 3.5 Data Extraction

Extracted data were organized into tables for easier comparative analysis (Higgins et al. 2019). The cognitive accessibility of the included instruments was assessed following the framework introduced by Kramer and Schwartz (2017). One of the authors reviewed each of the included instruments for each of the components related to cognitive accessibility within three design feature categories: content, layout and administration procedure. Scales were arranged according to whether the design features were explicitly chosen to match the needs of children with disabilities or not. Finally, each item from the included instruments was matched by one author to the dimensions and items of the MHC‐SF (Keyes 2006) to compare the underlying constructs.

## 4 Results

### 4.1 Data Extraction

**FIGURE 1:**
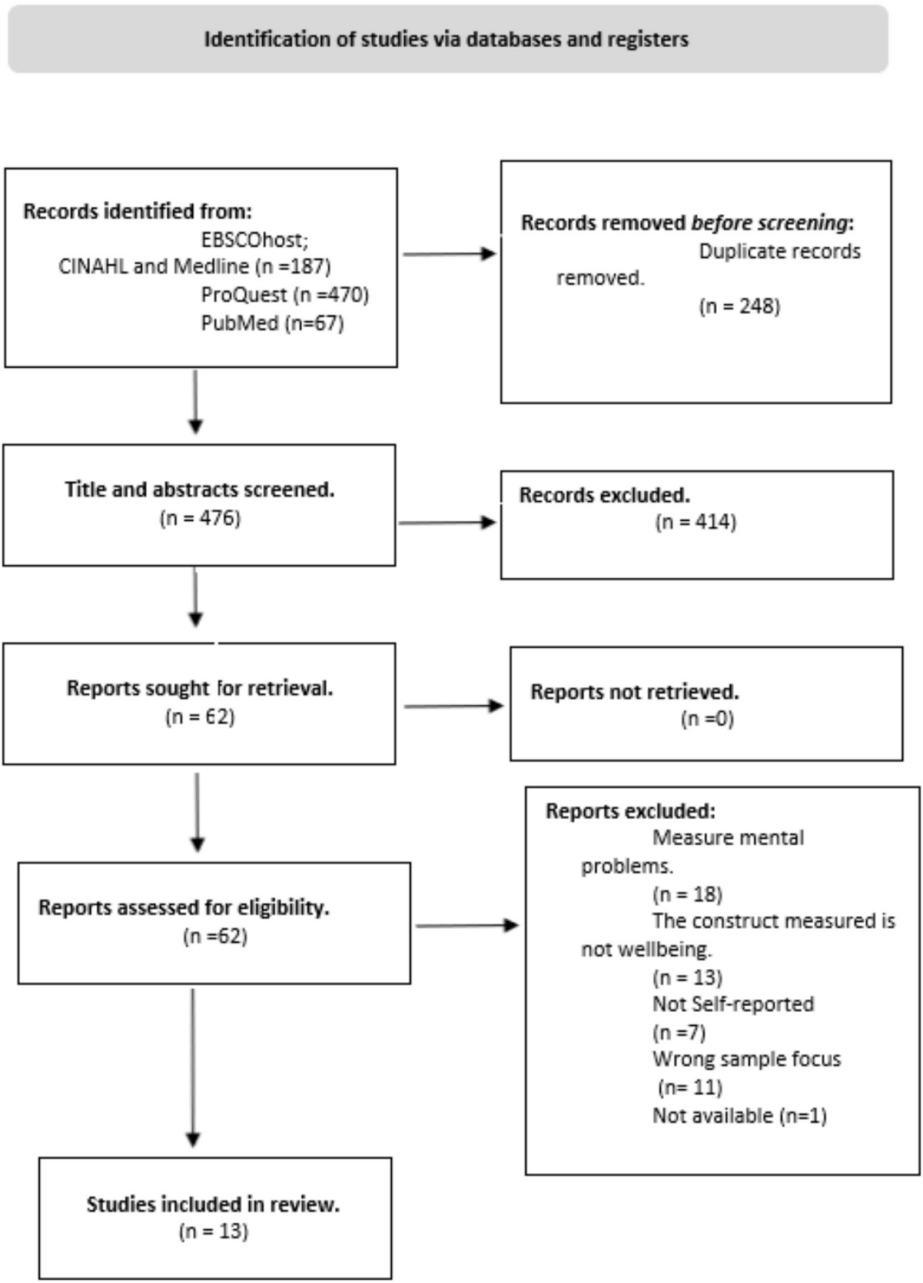
PRISMA flow chart of selected records.

A total of 476 unique records were identified after removing duplicates. The flow diagram following the PRISMA 2020 guidelines (Page et al. 2021) can be seen in Figure 1.

### 4.2 Instrument Overview

A total of 13 articles were included, in which 10 different instruments were used to measure well‐being. Two of the scales (the Kidscreen and the Warwick–Edinburgh Mental Well‐being Scale) appeared in three different versions and made up six of the 10 instruments. Six instruments were specifically designed for children with disabilities (see Table 2). Three measures were classified as measuring more than one dimension of well‐being, while eight (including different versions) were classified as unidimensional. An overview of the instruments extracted from the articles can be seen in Table 2.

**TABLE 2:** General overview of the included instruments.

Included instruments and the studies they were used in	The intended population of the instrument	The population studied in the included articles	Main concept and the total number of items in the scale	Subscales (number of items) or description of what it measures	Response scales	Quality assessment	Psychometric data retrieved from
Multidimensional measures							
MHC‐SF: (A) Skrzypiec et al. (2016); (B) Harris‐Lane et al. (2021)	Adolescents or adults	(A) SiSend, age 13–15 years (*N* = 172). (B) ADHD, age 15–24 years (*N* = 244)	Subjective well‐being (flourishing, languishing) Total items: 13	1. Emotional well‐being (2) 2. Psychological well‐being (6) 3. Social WB (5)	6‐level Likert‐type scale, frequency	Good	Lamers et al. (2010)
WellSEQ: (C) Boström and Broberg (2018); (D) Boström et al. (2018)	School‐age, mild/moderate intellectual disability	(C) Intellectual disability, age 13–16 years (*N* = 10). (D) Intellectual disability, age 12–16 years (*N* = 110)	Self‐rated mental health and ill‐health Total Items: 42	1. Subjective experiences of mental health (6) 2. Subjective experiences of mental ill‐health (13) 3. Peer relations and conflict (11) 4. School environment (6) 5. Family life (6)	3‐level Likert‐type scale, frequency (yes, sometimes, no)	Good	Boström et al. (2016)
Kidscreen‐27: Shearer et al. (2022)	School‐age, healthy or chronically ill	Cerebral palsy, age 8–18 years (*N* = 102)	HRQoL Total Items: 27	1. Physical activities and health (5) 2. General moods and feelings about yourself (7) 3. Family and free time (7) 4. Friends (4) 5. School and learning (4)	4‐level Likert‐type scale, quality 5‐level Likert‐type scale, agreement	Good	Ravens‐Sieberer et al. (2007)
Unidimensional measures							
Kidscreen‐10: Simões et al. ( 2015 )	School‐age, healthy or chronically ill	Special needs^a^, age 10–18 years (*N* = 472)	HRQoL Total items: 10	Can be used to assess subjective health and psychological, mental and social well‐being (HRQoL).	5‐level Likert‐type scale, frequency	Excellent	Ravens‐Sieberer et al. (2010)
ID‐Kidscreen‐10: Davison et al. (2022)	Intellectual disability	Intellectual disability, age 11–16 years (*N* = 35)			3‐level Likert‐type scale (yes, sometimes, no)	Good	Davison et al. (2023)
WEMWBS: (E) Houghton et al. (2022). (F) Harrowell et al. (2017)	General population	(E) NDD, age 10–16 years (*N* = 238). (F) DCD, age 17 years (*N* = 130)	Subjective well‐being Total items: 14	Measures hedonic and eudemonic well‐being elements.	5‐level Likert‐type scale, frequency	Excellent	Tennant et al. (2007)
WEMWBS‐SF: Sharpe et al. (2021)	General population	Different disabilities, age 12–25 years (*N* = 468)	Subjective well‐being Total items: 7			/	Tennant et al. (2007)^b^
ID‐WEMWBS: Davison et al. (2022)	People with intellectual disability	Intellectual disability, age 11–16 years (*N* = 35)	Subjective well‐being Total items: 7		3‐level Likert‐type scale, frequency (yes, sometimes, no)	Good	Davison et al. (2023)
Personal Wellbeing Index‐Cognitive Disability Scale (ID): Yousefi et al. (2013)	Intellectual disability	Intellectual disability, age 9–21 years (*N* = 200)	Personal well‐being Total items: 8	Measures of cognition (life satisfaction and subjective quality of life) and affect (positive affect).	10‐level Likert‐type scale (5–3–2–point alternatives)	Fair	Yousefi et al. (2013)
Subjective well‐being: Heiman and Olenik‐Shemesh (2020)	General population	Learning disability (*N* = 834)	Subjective well‐being Total items: 5	Global cognitive judgements of life satisfaction.	7‐level Likert‐type scale, agreement	Good	Diener et al. (1985)
WHO‐5 Well‐being Index: Warschburger et al. (2023)^c^	General population	Chronic conditions, age 12 years and older (*N* > 100)	Subjective well‐being Total items: 5	Subjective psychological well‐being based on positive mood, vitality and general interest.	5‐level Likert‐type scale	/	/

Abbreviations: ADHD, attention deficit hyperactivity disorder; DCD, developmental coordination disorder; HRQoL, health‐related quality of life; MHC‐SF, Mental Health Continuum Short form; NDD, neurodevelopmental disorder; SiSEND, self‐identified special education needs; WellSEQ, Well‐being in Special Education Questionnaire; WEMWBS, Warwick–Edinburgh Mental Well‐being Scale.

^a^ Special needs included chronic diseases, physical disability, a language or speech disability, learning disabilities and other non‐specified disabilities.

^b^ The same reference was provided by Sharpe et al. (2021) even though it did not provide psychometric data for the short‐form version.

^c^ This is a published study protocol and not an original article (thus no exact number of participants is reported).

Even though all included instruments measured well‐being in some way, the specific terms used varied (e.g., subjective well‐being, personal well‐being or self‐rated mental health). Furthermore, in some cases, there were inconsistencies in how the outcome of a scale was described across studies. Notably, quality of life measures were used to measure well‐being. For example, Davison et al. (2022) defined the Kidscreen‐10 as a subjective well‐being measure at first instance but switched to health‐related quality of life (HrQoL) later on.

The assessment using the COSMIN criteria revealed overall good to excellent psychometric properties of the studies. The Personal Wellbeing Index‐ID received the lowest quality rating of the included instruments. Although the internal and construct validity, as well as some aspects of reliability, were rated as good (Yousefi et al. 2013), no information was reported on test–retest reliability, prompting an overall score of fair according to the lowest rated‐box method (Mokkink et al. 2010). There were no psychometric studies from the WHO Well‐being Index referenced in the included paper that could be assessed; thus, it was not assessed for quality.

### 4.3 Cognitive Accessibility

An overview of the cognitive accessibility of the included instruments is depicted in Table 3. Basic layout aspects were not extensively described in the articles; however, based on the information at hand, there were many similarities in their design (consistency, item‐rating scale proximity and length of the text). When it comes to content accessibility, self‐reports made for children seem to adopt simple wording. Time restrictions for completing scales were generally unspecified; therefore, ‘self‐paced’ involves giving the choice to the respondent to focus and engage in one item as long as they wanted.

**TABLE 3:** Correspondence of the instruments to the cognitive accessibility factors.

Dimensions	Factors	Designed for children with disabilities (including health conditions)				Designed for the general population					
		WellSEQ	ID‐WEMWBS	ID‐Kidscreen‐10	Personal Wellbeing Index‐ID	Kidscreen‐10	Kidscreen‐27	MHC‐SF	Subjective Well‐Being Scale^a^	WHO‐5	WEMWBS
Content	Visual support	x	x	x							
	Grammar	x	x	x	x						
	Simple wording	x	x	x	x					x	x
	Currency	x	x	x					x		
	Construct examples	x	x	x							
	Questions	x	x	x	x	x	x				
	Self‐perception	x	x	x	x	x	x	x	x	x	x
	Positive wording		x						x	x	x
	Personal reference language								x	x	
Layout	Text adjacent to image	x	x	x							
	Short length text	x	x	x	x	x					x
	Consistency	x	x	x	x			x		x	x
	Item/rating visually close	x	x	x	x	x	x	x		x	x
Procedure	Self‐paced	x	x	x							
	Visual support	x	x	x	x						
	Reading alternatives	x	x	x	x			Cp assisted/assistant	Assistant		
	Response alternatives	x	x	x	x			Cp assisted	Assistant		

Abbreviation: Cp assisted: computer‐assisted personal interviewing.

^a^ The Subjective Well‐Being Scale (Diener 1985) is often used without presenting a digital or physical version of the scale to the informant; therefore, layout cannot be assessed.

The types and extent of adaptations in the instruments ranged from specific adaptations made by the authors in the administration procedures (e.g., use of an assistant to support reading comprehension in Skrzypiec et al. 2016; Heiman and Olenik‐Shemesh 2020) to design adaptations included in the characteristics of the instrument (e.g., adapting ID‐Kidscreen‐10 or ID‐WEMWBS using co‐design workshops by Davison et al. 2022). Instruments designed for children with disabilities addressed many accessibility aspects, particularly Davison et al. (2022) and Boström et al. (2016) who used participatory research methods with children with IDs.

The school context and in‐person were the most common characteristics of administration, following electronic questionnaires (Warschburger et al. 2023; Sharpe et al. 2021) and virtual meetings (Shearer et al. 2022). The WellSEQ (Boström et al. 2016) was the only instrument that pre‐established its administration through a supporting electronic tool (i.e., iPad).

Uniquely, the Personal Well‐being Index‐ID (Cummins and Lau 2004) favours accessibility by providing visual support in the response scale as well as response scale alternatives (10–5–3–2 point Likert). Harris‐Lane et al. (2021) used the data from the Community Health Survey—Mental Health (CCHS‐MH; Statistics Canada, 2013), which employed computer‐assisted personal interviews to administer the MHC‐SF.

### 4.4 Alignment With the Dual Continua Model and the MHC‐SF

**FIGURE 2:**
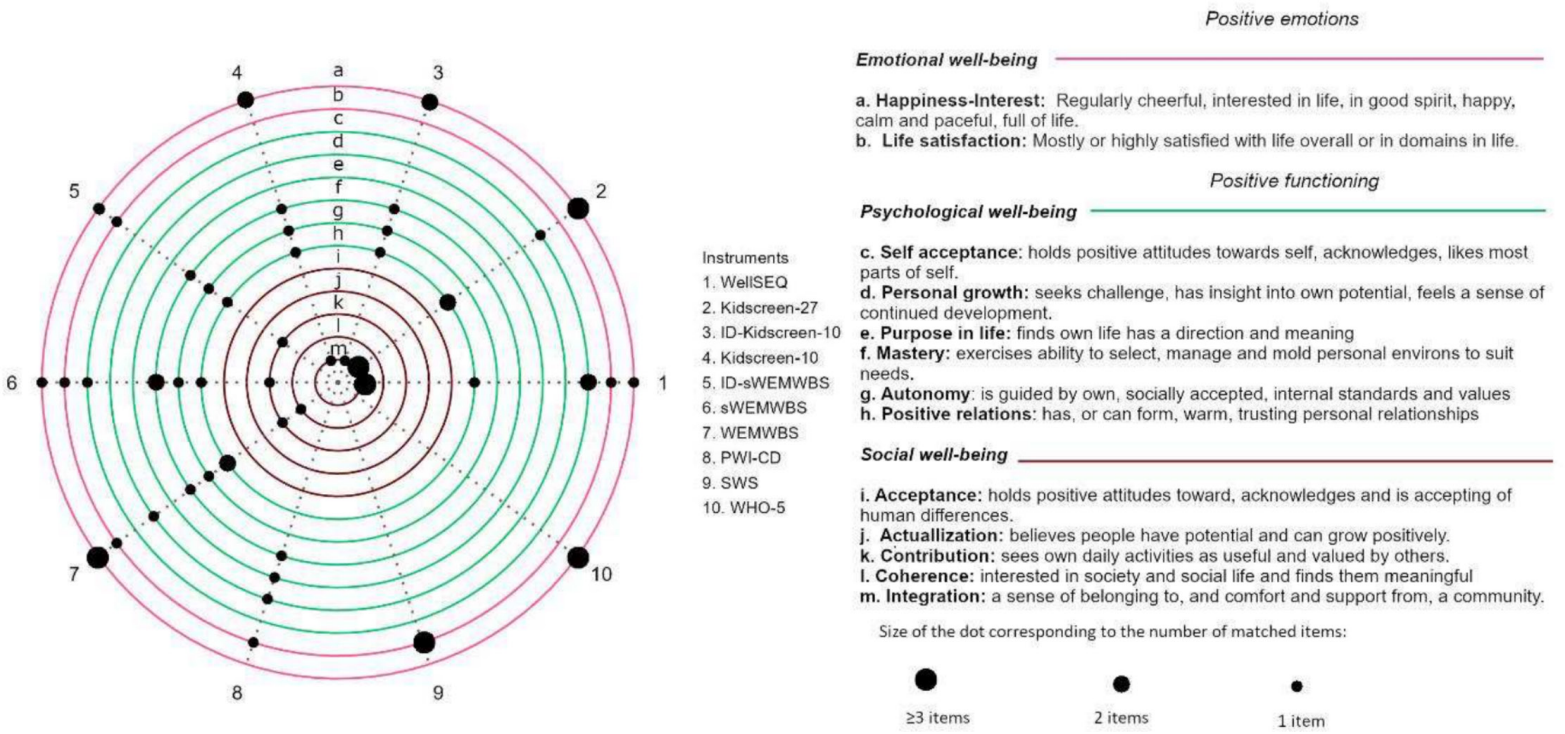
Correspondence between the instruments identified in the review (dotted lines) and the MHC‐SF dimensions (circles).

All three dimensions in Keyes' version of the dual continua model (as operationalized in the MHC‐SF) were represented in the included instruments to some degree (see Figure 2 for a visualization of the overlap). Social well‐being (positive social functioning) was the dimension least covered by the instruments, while emotional well‐being (positive emotions) was the most frequently occurring dimension. The WHO‐5 and Subjective well‐being (Diener 1985) matched entirely with the MHC‐SF emotional well‐being items measuring happiness and life satisfaction.

The WEMWBS (in one or the other version) contained items matching all the items of the psychological well‐being scale of the MHC‐SF, except for Purpose in life. Personal growth and Purpose in life were the aspects of psychological well‐being (i.e., Personal well‐being index‐Cognitive disability ‘The things you make or the things you learn’; ‘how things will be later in your life?’) that were least represented in the included scales. Contrary, Self‐acceptance (e.g., ‘Have you been happy with the way you are?’ from Kidscreen‐27), Environmental mastery (e.g., ‘I have been dealing with problems well’ from WEMWBS) and Autonomy (e.g., ‘Have you made your own choices today?’ from ID‐WEMWBS) were more frequently represented.

Although Positive relations was the most common aspect of psychological well‐being in the included instruments, the matching item's meaning tended to overlap with the social Well‐being dimension Integration. Some items could not be clearly distinguished from each other given that they contained both individual connotations from Positive relations and community connotations from Integration (e.g., ‘Do you feel lonely?’ from WellSEQ and Kidscreen and ‘Have you and your friends helped each other?’ from Kidscreen‐27). Negatively worded items primarily occurred in the social well‐being item Integration (e.g., absence of conflict or loneliness). The other dimensions of social well‐being were only represented by the WEMWBS measures, having Coherence (e.g., ‘I have been feeling useful’ Contribution; ‘I have been feeling interested in other people’ Coherence) only in the full version.

## 5 Discussion

We conducted a scoping review of the self‐report instruments used to measure well‐being in children with disabilities and assessed their psychometric properties, cognitive accessibility and the convergence of the instruments with the dimensions of well‐being proposed by Keyes et al. (2002). Ten self‐report instruments used in studies involving children with disabilities were identified with fair to excellent psychometric properties. Aspects of cognitive accessibility relating to relevant design domains (i.e., content, layout and procedure; Kramer and Schwartz 2017) were identified across studies. The instruments with the most examples of such specific design features were WellSEQ, ID‐WEMWES and ID‐Kidscreen‐10. Moreover, the assessment of the underlying dimensions showed that instruments are primarily focused on emotional well‐being and less so on social well‐being. A more comprehensive operationalization of well‐being using the three dimensions (i.e., emotional, psychological as well as social) was rarely reflected by the instruments.

### 5.1 Instruments Cognitive Accessibility

Measures of well‐being used with children with disabilities use various design features to increase cognitive accessibility. The accounts made by instrument developers largely matched those listed in the framework outlined by Kramer and Schwartz (2017). Assistants are commonly used to provide motor and cognitive support; perhaps because flexibility in administration procedures is viewed as a weaker threat to validity than modifications to material and/or layout. Such flexibility might open a door to inclusion, as discussed by Davison et al. (2022). Formats such as autonomous interactive digital versions, like the one applied in WellSEQ, could be a way to include children with disabilities in large‐scale surveys where in‐person interviewing is not a feasible option.

The most common adaptations concerned consistency, item‐rating scale proximity and length of the text. These design features did not seem to be related to whether the target group was children with disabilities or not. However, when explicitly adapting instruments to the needs of children with disabilities, the adjustments made were often chosen in collaboration with children with disabilities. These adaptations included a wider range of design features, especially regarding content (e.g., use of construct examples and time currency) and administration procedures (e.g., reading and listening alternatives). One way of involving the perceptions of the children in the design of measures was through the use of focus groups in the construction phase. Including the perspective of children with disabilities can be expected to increase ecological validity, which is related to the aspects of accessibility proposed by Kramer and Schwartz (2017).

### 5.2 Instrument Dimensions

In this study, the relation between the identified instruments and Keyes' version of the dual continua model (Keyes et al. 2002) was done by comparing items from the instruments with the dimensions and items in the MHC‐SF. Results revealed that social well‐being was the least frequently occurring dimension in the reviewed instruments. This can partly be explained by the fact that items covering social well‐being seem to overlap with items designed to measure aspects of psychological well‐being. For example, the most prevalent social well‐being dimension, ‘Integration’ (e.g., ‘Have you felt lonely?’), overlaps with the psychological well‐being dimension ‘Positive relations’. The level of overlap depends on the level of abstraction of the item from an individual to a community focus. Joshanloo and Nosratabadi (2009) pointed out that the MHC‐SF portrays well‐being as a primarily private phenomenon. Consequently, individual aspects of well‐being are emphasized in the measures identified in the present review. For example, ‘feeling helpful’ (e.g., the item ‘Do you feel helpful today’ from WEMWBS) may relate to self‐acceptance (Greenfield 2009) rather than social aspects (e.g., sense of belonging) highlighting the interconnectedness of individuals with their environment. A strong focus on the individual level may limit the concept of positive functioning and neglect how we are embedded in social structures and communities.

The identified multidimensional measures for children with disabilities used family, school and peers to contextualize well‐being. An alternative approach to producing relevant concrete examples and contexts for social well‐being for children with disabilities could be to involve children in the process through participatory research methods. By asking the children questions regarding ‘feeling useful’, other dimensions of social well‐being can be elaborated. Slowly but surely, literature on children's perspectives is growing as they are recognized as social actors who play a role in structuring their childhood (Mason and Hood 2011). The review at hand provided interesting examples of such approaches, for example by introducing the notion of ‘feeling helpful’ (e.g., ‘Do you feel helpful today’ from WEMWBS) related to purpose or self‐acceptance (Greenfield 2009).

### 5.3 Quality of Life or Well‐Being

Five out of 10 identified measures were termed quality of life measures by their developers, including the variations of the Kidscreen. Items from these measures could primarily be matched to emotional and psychological well‐being. Other measures of quality of life, such as the personal well‐being index (Cummins and Lau 2004) and the WHO‐5 (Topp et al. 2015), focused on emotional well‐being. Some quality‐of‐life measures focus primarily on limitations in performing activities (Taillefer et al. 2003) and might therefore be of limited value when describing positive aspects of functioning. Worth noting is that in Keyes' (2002) description of the construction of the MHC‐SF, Cantril's (1965) self‐anchoring item is mentioned, but only as an example of positive affect and not a standalone item. In subjective well‐being, the individuals' evaluations of the quality of their lives and life functioning are separate dimensions (Keyes et al. 2002) and quality of life and functioning are only weakly correlated (Shelly et al. 2008; Davis et al. 2013). This indicates that functioning and emotional well‐being might be fairly independent dimensions of well‐being that both need to be measured as illustrated by Keyes' MHC‐SF.

### 5.4 Construct of Well‐Being

There are inconsistencies in the conceptualization and definitions of well‐being (e.g., Diener and Seligman 2004) that were evident in the instruments identified in the review. The equation of subjective well‐being with emotional well‐being (i.e., as represented in the hedonic tradition) might be linked to the use of the concept in quality‐of‐life theories. Eudemonic well‐being is typically operationalized as positive functioning referring to psychological well‐being. Nevertheless, there is growing agreement that hedonic and eudemonic well‐being are two aspects that both need to be represented in a measure of mental health operationalized as well‐being (e.g., Keyes 2002; Tennant et al. 2007; Seligman 2011; Diener et al. 2010; Allin and Hand 2017). By including both, aspects of emotional well‐being as well as psychological and social functioning are represented.

Diener (1985) originally developed the Subjective Well‐Being Scale only considering hedonic aspects of well‐being. At a later stage, he enriched his measure by adding the construct of ‘flourishing’, including the components of purpose in life, positive relationships, engagement, competence, self‐esteem, optimism and contributions towards the well‐being of others (Diener et al. 2010). Keyes' (2002) proposal of global dimensions of psychological and social well‐being is unique in the sense that these dimensions are often used deconstructed, meaning well‐being is measured in independent pieces rather than a global integration representing the whole construct of well‐being. For example, the WHO uses subjective well‐being in its hedonic definition (i.e., WHO‐5) while acknowledging it as a part of mental health concepts such as self‐efficacy, autonomy, competence and self‐actualization (WHO 2001). The overlaps, especially within eudemonic well‐being, and psychological and social well‐being (Stewart‐Brown et al. 2009; Young et al. 2023) further strengthen the argument for a global measure. Greater attention to the overlap can lead us to establish the shared operative processes and the shared variance in optimal functioning (Snyder and Lopez 2002).

## 6 Limitations

The relatively few identified records could be seen as a sign that the search strategy was not optimal. However, in a recent systematic review of instruments used for mental health problems in children with an ID, the same number of instruments were identified (Halvorsen et al. 2022), even though problem‐focused outcomes tend to retrieve more attention than positive outcomes. The lack of research on appropriate indicators of well‐being has been previously pointed out (e.g., Llewellyn and Leonard 2010; Huebner et al. 2002), and the relative sparsity of instruments could be an expected consequence of the search being dedicated specifically to positive mental health or well‐being, excluding quality of life. It is noteworthy that while the selection process was conducted by two researchers, the data extraction and the quality assessment were performed solely by the first author. Lastly, the framework by Kramer and Schwartz (2017) was used to assess cognitive accessibility in the included instruments, even though it was not developed for that purpose. Consequently, it was only possible to estimate the number of different types of adaptations used and not the overall level of cognitive accessibility in the scales. Through this framework, we placed the focus on the cognitive accessibility of the instruments; therefore, cognitive profiles of participants were limited to the reporting, offering a static view instead of exploring the dynamism of the concept.

## 7 Conclusion

In this scoping review, self‐reported well‐being measures for children with disabilities were examined, revealing their increasing inclusion in research. Despite this progress, there remains a lack of consensus on a comprehensive self‐report measure for well‐being. The instruments reviewed aligned strongly with Keyes's (2002) hedonic (emotional) and eudemonic (psychological and social) dimensions, indicating that Keyes's model is a robust framework for understanding well‐being. However, well‐being is primarily portrayed as an individual phenomenon leading to an under‐representation of social well‐being. Recognizing the social aspect of well‐being is crucial, particularly for stigmatized groups. Potentially, low scores on social well‐being may indicate low perceived inclusion.

To develop appropriate measures, focusing on central aspects of a child's life—such as school, family and peers—is essential. Children's perceptions should guide the creation of useful items within these contexts. Traditional psychometric validation may not suffice to ensure the accessibility of scales. Participatory research methods can provide valuable insight into layout, administration and construct accessibility, which are not achievable through conventional approaches. Additionally, digital tools might enhance inclusion in large‐scale self‐report surveys. Achieving a comprehensive and accessible well‐being measure for children with disabilities requires ongoing collaboration, innovative methodologies and a commitment to amplifying the voices of those directly affected.

## Author Contributions

**Estrella Torres Cabo:** writing – original draft, writing – review and editing. **Mats Granlund:** writing – review and editing. **Magnus Ivarsson:** writing – review and editing.

## Conflicts of Interest

The authors declare no conflicts of interest.

## Data Availability

The data that support the findings of this study are openly available in the repository in OSF with the same title as this article at this link, DOI 10.17605/OSF.IO/VX2TG. Missing data are available upon reasonable request. *[Dataset]* Cabo, E. T., Ivarsson, M., & Granlund, M. (2024, May 28). Mapping the instruments used to measure well‐being on children with disabilities: A scoping review. Retrieved from osf.io/vx2tg
